# Deanxit and tandospirone relieved unexplained limb edema in a depressed pituitary adenoma survivor: A case report

**DOI:** 10.3389/fpsyt.2022.965495

**Published:** 2022-11-10

**Authors:** Xingyu Dong, Sheng Fang, Wei Li, Xuemei Li, Sunfu Zhang

**Affiliations:** ^1^Department of Neurosurgery, The Third People's Hospital of Chengdu, Chengdu, China; ^2^Department of Neurology, The Third People's Hospital of Chengdu, Chengdu, China

**Keywords:** limbs edema, Deanxit and tandospirone, major depressive disorder, somatic symptoms, antidepressant

## Abstract

Our case report describes a 45-year-old woman who suffered from limb edema for 2 months. We focused on tumor recurrence and other common potential diseases based on the pituitary adenoma history. However, none of the examinations showed any abnormality. Later, her continuous complaints about the family relationship and depressed mood came into sight, and a psychiatry consultation was arranged. Following that, she was diagnosed with major depressive disorder. After several days of Deanxit and tandospirone treatment, the patient's limb edema dramatically subsided. This is the first case of limb edema associated with depression. This highlights the importance of awareness of mental illness for non-psychiatrists, especially in patients with severe somatic symptoms, but with negative results.

## Introduction

Edema is an accumulation of fluid in the interstitial space that occurs as the capillary filtration exceeds the limits of lymphatic drainage (1). It is usually seen in the eyelid, the infraorbital soft tissue, and the limbs, especially in the lower extremities. Edema can be caused by many somatic conditions, including endocrine and metabolic disease, cardiac disease, hepatic disease, renal disease, venous disease, and unspecific reasons (1, 2). Depression is a psychiatric condition associated with stratified psychological and somatic symptoms (3). Somatic symptoms prevailed in most patients with depression (4). Somatic symptoms vary from person to person. The most frequently presented ones are chronic pain, fatigue, and sleep disturbance. Limb edema associated with depression is rare. Herein, we report a case of a 45-year-old woman who had limb edema and was cured using Deanxit and tandospirone.

## Case description

The patient suffered from limb edema for 2 months. She had pituitary adenoma for 7 years and underwent transsphenoidal pituitary surgery 4 years ago. She went to the hospital for an endocrine review 2 years, which were within normal ranges.

Upon initial assessment, we observed mild to moderate edema in her hands and lower extremities, which the patient had complained about. She stated that she could not clench her fists and had recently felt that her pants were getting tight. However, her weight was stable from morning to evening. In addition to limb edema, she complained about feeling fatigued, depressed, and anxious and about having irregular menstruation in the last 2 months. The changes in her mental status and behavior prevented her from getting along with her family. Her vital sign and other physical examination were normal as well.

## Diagnostic assessment and treatment

Considering her pituitary adenoma history and limb edema, we considered pituitary adenoma recurrence and secondary hypothyroidism as our initial diagnosis. Therefore, we scheduled a brain MRI with pituitary protocol and endocrine examination. However, the endocrine examination returned nearly normal results. Her thyroid hormone, sex hormone, growth hormone, and cortisol levels were within normal ranges. Moreover, the brain MRI only showed a slight enhancement in the pituitary gland, which was insufficient to diagnose tumor recurrence. As a result, the patient underwent further examinations, including liver and renal function tests, cardiac color Doppler ultrasonography, and internal organ ultrasonography. However, none of them displayed any abnormality. Thus, the common causes of limb edema, such as hypoalbuminemia, heart failure, renal failure, venous insufficiency, and idiopathic edema, were excluded.

Her continuous complaints, including about her daily life and family relationship, caught our attention on the fourth day of admission. Therefore, the psychiatry consultation was arranged immediately. At first, our psychiatry team performed PHQ-9, GAD-7, and Life Event Scale (LES) on her. The patient scored 20 in PHQ-9, 20 in GAD-7, and 65 in LES. These results indicated that the patient suffered severe mood problems in her daily life. The Hamilton Anxiety Rating Scale (HAMA) score was 25, and the Hamilton Depression Rating Scale (HAMD) score was 22. She was diagnosed with major depressive disorder.

The treatment that was given to her consisted of Deanxit (flupentixol/melitracen) 10.5 mg b.i.d. and tandospirone 10 mg t.i.d. The patient showed a significant improvement after 7 days of treatment. She felt much more relaxed. In addition, edema was relieved significantly. Then, she was discharged and referred to an outpatient psychiatric consultation with an indication for maintenance antipsychotic medication.

**Figure 1:**
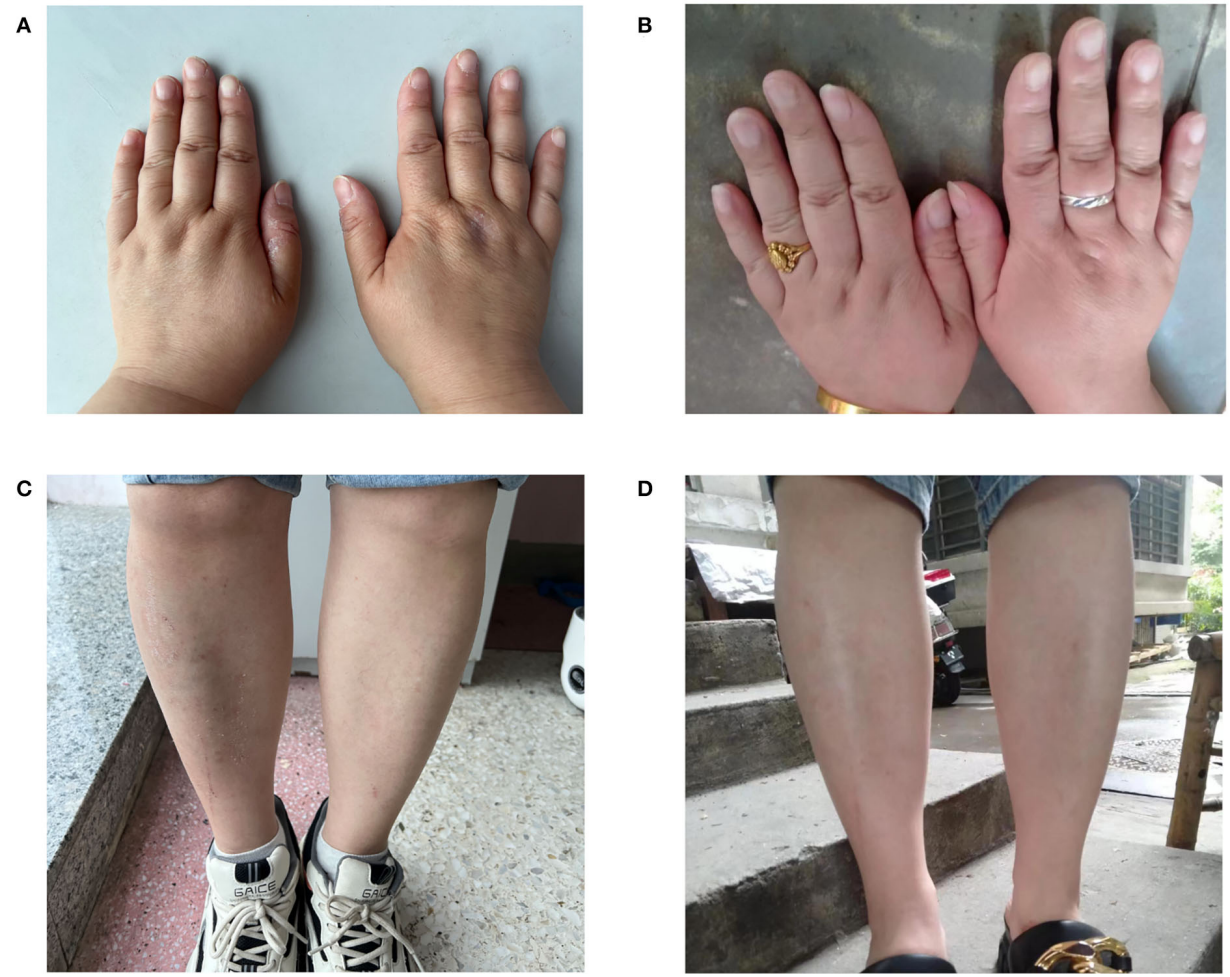
Limbs edema relived after antidepressant therapy at 7 days **(A,C)** and 3 weeks **(B,D)**.

At the 3-week follow-up, the patient mentioned that she could wear her ring again (Figure 1). At the 2-month follow-up, her psychiatric symptoms further recovered. The HAMA and HADM scores were 8 and 9, respectively. The symptoms such as fatigue, depression, anxiety, and upset disappeared. In addition, she started to engage in more social activities, and her relations with family members improved. No relapse of psychiatric or edema was detected.

## Discussion

Chronic limb edema is usually caused by systemic diseases like heart failure, renal disease, hepatic disease, or endocrine disease. The most frequent type is pitting edema, while myxedema is rare and often seen in Graves' disease and hypothyroidism. The thyroid hormone plays an essential role in many bodily functions. The extreme absence of this hormone is associated with a high mortality rate and has a broad spectrum of presenting symptoms (5). Thyroid hormone has several roles within the central and peripheral nervous systems, explaining the varying degrees of neurological symptoms associated with hypothyroidism, such as depression (6). Patients who underwent pituitary surgeries often suffer from hypopituitarism, which leads to secondary hypothyroidism. In addition to bilateral limb edema, this patient had a history of pituitary surgery and did not take hormone replacement therapy after the surgery. As a result, we suspected hypothyroidism to be the cause of limb edema and psychiatric symptoms. However, the levels of thyroid-stimulating hormone (TSH), triiodothyronine (T3), and thyroxine (T4) of the patient were normal and negated our initial diagnosis.

The other common type of edema we need to differentiate is idiopathic edema. *Idiopathic edema* is a state that primarily affects women in the absence of hepatic, renal, and cardiac diseases (7–9). The diagnosis is made by physical and clinical assessments, and diuretics are the common treatment (10). The hallmark of this disease is orthostatic edema, accompanied by excessive weight gain from morning to evening due to fluid retention. It occurs almost exclusively in post-pubertal women and is independent of the menstrual cycle (11). However, in our case, the patient's weight was stable from morning to evening. She did not have orthostatic edema either. The clinical presentations are insufficient for the diagnosis of idiopathic edema.

Depression is a highly heterogeneous disorder with complicated clinical manifestations and biological mechanisms (12, 13). Two-thirds of patients with depression present only somatic symptoms at the early stage, and approximately half of the patients have unexplained medical symptoms (14). Sometimes lymphedema is accompanied by high depression and anxiety (15). However, unlike our case, this type of edema is usually caused by malignant tumors. In addition, some patients with idiopathic edema were more depressed and anxious, with a trend toward widespread neurotic symptoms (16). In these circumstances, diuretics are widely used to relieve edema, which might also help release psychiatric symptoms. However, concurrent psychiatric symptoms were less recognized and treated.

Some researchers believed that excessive fluid retention in many patients with idiopathic edema might be related to the neuroendocrine abnormalities associated with psychiatric illness (16). We encountered similar situations in our case, and we discovered that edema was relieved at the same pace as her mood symptoms. However, there were hardly any published work suggesting Deanxit or tandospirone usage in such cases.

Moreover, neither of these medications has a diuretic effect. This evidence indicates that Deanxit and tandospirone do not relieve edema through a diuretic effect. We speculated that our patient had severe psychiatric problems at first. Then, primary emotional disturbances initiated some neuroendocrine changes and led to limb edema. Our treatment with Deanxit and tandospirone relieved depression, fixed neuroendocrine abnormalities, and reduced the limb edema. Our case might be the first report that limb edema, most likely associated with depression, dramatically remitted after antidepressant treatment. The underlying mechanism of this phenomenon is unknown, and further studies are needed.

People often present with somatic symptoms to clinical settings (17). Some non-specific symptoms may overlap with the somatic symptoms of psychiatric disorders, leading to misdiagnosis. Moreover, somatic symptoms could double the medical cost of examinations and treatment (18). As a result, physicians should consider psychiatric disorders when unexplainable symptoms appear. After treating limb edema, we turned to the psychiatrist for help as soon as we excluded the physical disorder of the patient, and the prescribed antidepressants finally solved the patient's problem.

## Data availability statement

The raw data supporting the conclusions of this article will be made available by the authors, without undue reservation.

## Ethics statement

Written informed consent was obtained from a legally authorized representative(s) for anonymized patient information to be published in this article.

## Author contributions

XD and SF drafted this manuscript and treated the patient. WL conducted a psychological analysis. SZ and XL performed the case analysis. All authors contributed to the article and approved the submitted version.

## Funding

This work was supported by the Sichuan Science and Technology Department (2021YFS0082) and Chengdu Science and Technology Bureau (ZX20210805420).

## Conflict of interest

The authors declare that the research was conducted in the absence of any commercial or financial relationships that could be construed as a potential conflict of interest.

## Publisher's note

All claims expressed in this article are solely those of the authors and do not necessarily represent those of their affiliated organizations, or those of the publisher, the editors and the reviewers. Any product that may be evaluated in this article, or claim that may be made by its manufacturer, is not guaranteed or endorsed by the publisher.
